# The Impact of CT Imaging on the Diagnosis of Fragility Fractures of the Pelvis: An Observational Prospective Multicenter Study

**DOI:** 10.3390/jcm15020531

**Published:** 2026-01-09

**Authors:** Michał Kułakowski, Karol Elster, Wojciech Iluk, Dawid Pacek, Tomasz Gieroba, Michał Wojciechowski, Łukasz Pruffer, Magdalena Krupka, Jarosław Witkowski, Magdalena Grzonkowska, Mariusz Baumgart

**Affiliations:** 1 Orthopedic and Trauma Department, Jan Biziel University Hospital nr 2, 85-168 Bydgoszcz, Poland; michwoj89@gmail.com; 2 Faculty of Health Sciences, Powislanski University, 82-500 Kwidzyn, Poland; 3 Department of Orthopaedic and Trauma Surgery, Provincial Integrated Hospital, 82-300 Elbląg, Poland; 4 Department of Orthopaedics and Traumatology of the Musculoskeletal System, 109 Military Hospital, 71-422 Szczecin, Poland; pacek1984@gmail.com (D.P.); lukasz.pruffer@gmail.com (Ł.P.); 5 Department of Trauma and Orthopaedic Surgery, Regional Specialistic Hospital, 20-718 Lublin, Poland; tomgieroba@gmail.com (T.G.); magdalenakrupka@interia.eu (M.K.); 6 Department of Orthopedics, Traumatology and Hand Surgery, Faculty of Medicine, Wroclaw Medical University, 50-367 Wroclaw, Poland; jaroslaw.witkowski@umw.edu.pl; 7 Department of Normal Anatomy, The Ludwik Rydygier Collegium Medicum in Bydgoszcz, The Nicolaus Copernicus University, 87-100 Toruń, Poland; m.grzonkowska@cm.umk.pl (M.G.);

**Keywords:** fractures, pelvis, computed tomography

## Abstract

**Background/Objectives**: Fragility fractures of the pelvis (FFPs) are a significant concern in the elderly population, often leading to severe morbidity and mortality. This study aims to evaluate the diagnostic challenges, clinical outcomes, and mortality rates associated with FFPs in patients referred to multiple hospitals. **Methods**: A total of 99 patients with suspected pelvic fragility fractures were enrolled between January 2023 and June 2025. Initial diagnoses were made using plain X-rays, with computed tomography (CT) utilized to assess posterior ring fractures. Data on demographics, fracture types according to the Fragility Fracture of the Pelvis (FFP) Classification, hemoglobin levels, and mortality rates were collected and analyzed. **Results**: The findings revealed that while plain X-rays identified only anterior pelvic ring fractures, CT scans detected posterior ring fractures in 60.6% of cases. Patients with Nakatani II and III pelvic ramus fractures exhibited the most significant decreases in hemoglobin levels. The overall mortality rate was found to be 13.13%, with the highest rates observed in FFP I (13.5%) and FFP II (11.9%) groups. **Conclusions**: The findings of this study underscore the importance of CT imaging in the diagnosis of FFPs and highlight the need for close monitoring of hemoglobin levels in affected patients. This study also emphasizes the increased mortality risk associated with more complex fracture types. Future research should focus on evaluating functional independence and treatment outcomes to guide clinical decision-making in managing fragility fractures of the pelvis.

## 1. Introduction

Fragility fractures represent an increasing problem in contemporary society [1,2,3] and are associated with an increased risk of mortality [4,5]. Systematic reviews have reported a 1-year mortality of 21.8% for hip fractures, 20.5–27% for vertebral fractures [6], and 15.5% for fragility fractures of the pelvis [7].

Due to reduced bone density, fragility fractures of the pelvis (FFPs) predominantly affect older individuals [8,9]. The incidence of these life-threatening fractures increases significantly with age and affects, in particular, women over 65 years old [10].

Among life-threatening fractures, fragility fractures of the pelvis (FFPs) remain poorly understood. In recent decades, their prevalence has risen steadily, reflecting the growing ageing population. In fact, FFPs are one of the main causes of loss of independence and decreased quality of life.

The characteristics of FFPs, in particular, their trauma mechanisms and presenting symptoms, differ from those observed in younger trauma patients. Treatment algorithms are still not clear, with some authors arguing for conservative treatment and others for operative treatment [11,12,13].

There are few studies and no prospective trials concerning mortality associated with FFPs and treatment algorithms [14,15]. A study published by Rommens and Hoffman [16] presented treatment algorithms for FFPs, which are indications for operative treatment in the case of instability. Operatively treated patients stay longer in the hospital, which increases the risk of complications, but Wagner et al. [12] reported lower mortality rates and better mobility after operative treatment. Operative treatment may also be associated with a higher complication rate due to the risk of neurovascular complications associated with the particularly complicated pelvic anatomy [17].

Moreover, the incidence of posterior ring fractures among patients presenting pubic fractures observed in plain X-rays is not clear in the literature, with reported values ranging from 12% to 87% [16,17,18,19].

The aim of our study is to evaluate the incidence of posterior ring fractures in computed tomography among older patients presenting anterior ring fractures in plain X-rays.

A secondary objective is to evaluate the mortality rate and blood loss in particular groups of patients with FFPs and to identify the factors that influence decision-making and outcome.

## 2. Materials and Methods

This study was conducted in accordance with the Declaration of Helsinki and approved by the bioethical committee of the Kujavian-Pomeranian Local Medical Chamber.

Demographic and medical history data of all patients referred with pubic fractures observed in plain X-rays between January 2023 and June 2025 were prospectively collected. All patients over 65 years old were diagnosed by computed tomography in order to examine the incidence of posterior ring fracture.

This was a multicenter study performed between January 2024 and June 2025 at the Clinical Department of Orthopaedics and Trauma, Jan Biziel University Hospital nr 2 in Bydgoszcz; the Department of Trauma and Orthopaedic Surgery, Regional Specialistic Hospital in Lublin; the Department of Orthopaedics and Traumatology of the Musculoskeletal System, 109 Military Hospital in Szczecin; and the Department of Orthopaedic and Trauma Surgery, Provincial Integrated Hospital in Elbląg.

All patients referred to the aforementioned hospitals with pelvic pain after minor trauma were diagnosed. The inclusion criteria were pubic fractures after minor trauma in patients over 65. Age was collected as a continuous variable. No predefined age cut-off was used to define subgroups, because fragility fractures were defined primarily by low-energy mechanism/osteoporosis rather than an arbitrary age threshold. Diagnosis was made by means of anamnesis, plain X-rays, and computed tomography (CT). All patients were classified according to the Fragility Fractures of the Pelvis (FFP) Classification [20].

All patients younger than 65, with high-energy trauma and pathological fractures, were excluded.

The following data were collected: age, sex, comorbidities, date of admission, days of stay, FFP Classification, treatment (operatively vs. conservatively), hemoglobin level at admission and after one day, and Nakatani classification of pubic ramus fracture [21], as well as 30-day and 90-day mortality.

A comorbidity was defined as a known disease with which the patient was admitted. Recommendations published by Rommens and Hoffmann were the source of further treatment [22,23]. Patients with FFP I and II were treated conservatively, and those with FFP III and IV were treated operatively.

Patients or their relatives were contacted by phone 30 and 90 days after admission. All collected personal data were anonymized. All patients or their relatives gave written approval for participation in the study.

### Statistical Analysis

The normal distribution of numerous data was tested using the Kolmogorov–Smirnov test. Descriptive statistics were used to describe the study population, using mean and standard deviation (SD) for normally distributed data. If the data were not normally distributed, the median and interquartile range (IQR) were utilized.

The level of significance was defined at *p* < 0.05. The data were analyzed using Statistica12.5 software.

## 3. Results

All patients referred between January 2023 and June 2025 with suspected fragility fracture of the pelvis to the aforementioned hospitals were enrolled in the study. All patients had a plain X-ray of the pelvis performed, and if the anterior pelvic ring was fractured, a CT scan was performed.

**Figure 1:**
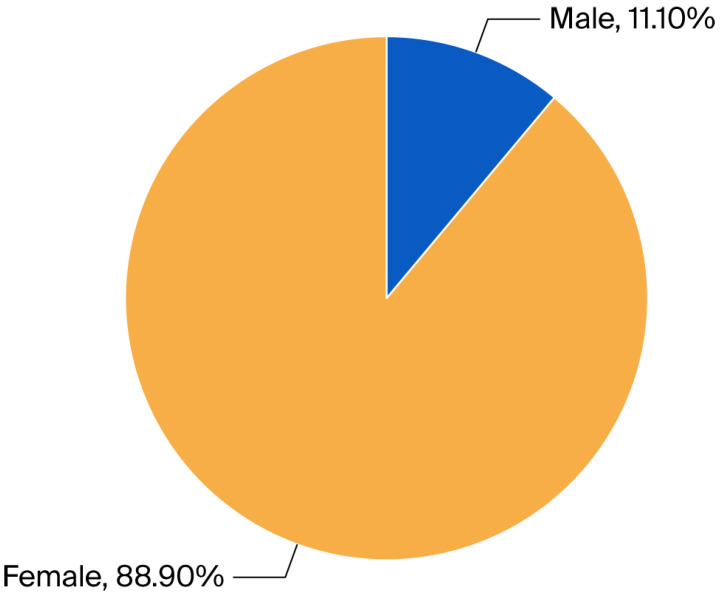
Gender distribution in %.

In total, 99 patients were enrolled; 88 (88.9%) of them were female and 11 (11.1%) male. Mean age was 81.12 with SD 10.13 (Figure 1). Patients stayed in the hospital between 1 and 30 days (mean 4.86; SD = 4.48).

**Figure 2:**
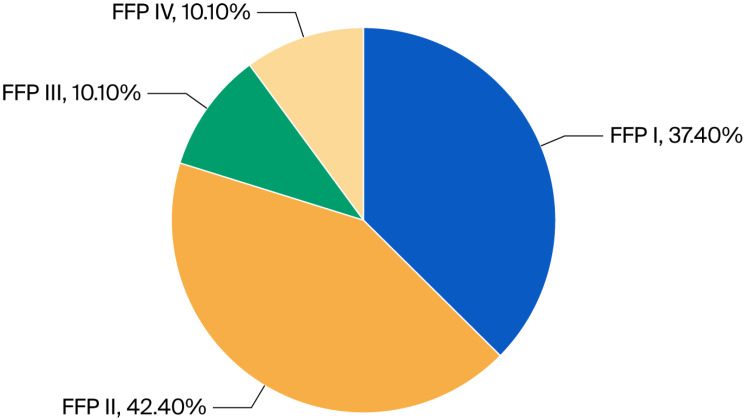
Distribution of FFP types in %.

**Figure 3:**
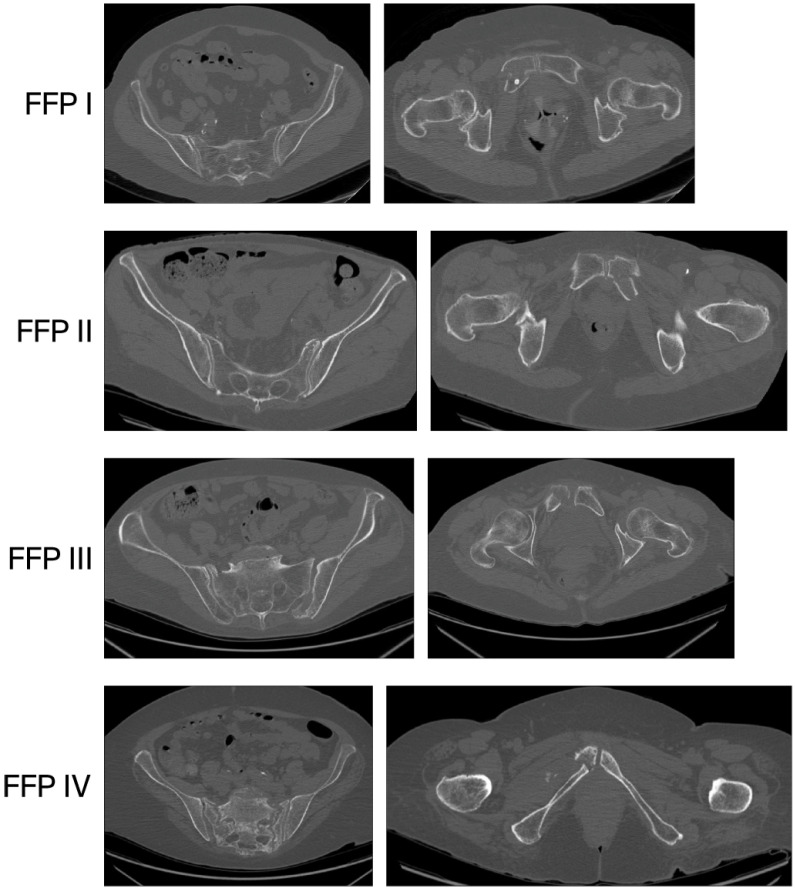
FFP types.

Every patient was examined with CT to assess the posterior pelvic ring, and in 62 (60.6%) cases, the posterior ring was fractured. According to the aforementioned Fragility Fracture of the Pelvis Classification, 37 (37.4%) fractures were assessed as type I, 42 (42.4%) as type II, 10 (10.1%) as type III, and 10 (10.1%) as type IV (Figure 2 and Figure 3).

All patients underwent laboratory tests during admission and the day after, and a decreased hemoglobin level of 2 g% or over was detected in 20 (20.2%) patients. According to the Nakatani classification of pubic fractures, the greatest decrease was detected in Nakatani II (8–8.08%) and III (11–11.1%), and only in three (3.03%) cases in Nakatani I. Altogether, hemoglobin levels greater than 2 g% were detected in 19.18% of Nakatani types I and II. Hemoglobin drop could also result from the administration of anticoagulants. In our cohort, 47 (47.4%) patients were administered NOACs (novel oral anticoagulants) or VKAs (vitamin K antagonists). We did not find a correlation between administration of the aforementioned drugs and Hb drop (*p* > 0.05).

According to the FFP classification, the decrease in HB levels was as follows: I—11.1%; II—7.07%; III—4.04%.

**Figure 4:**
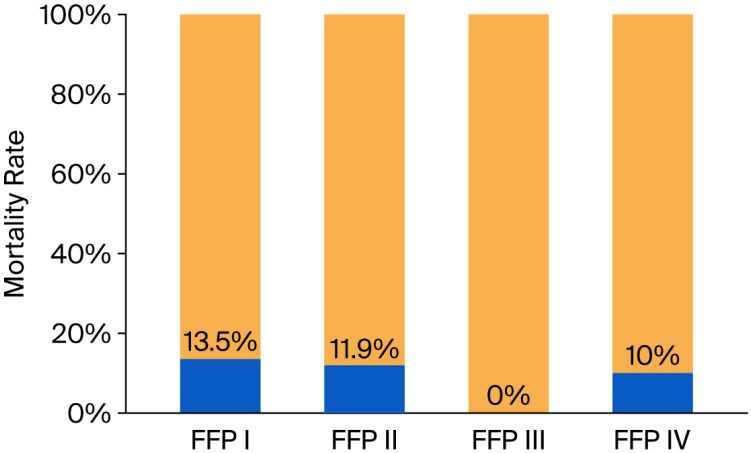
Mortality rate in %. *p* = 0.681.

The authors also assessed the mortality rate. Overall mortality rate was 13.13%, 30-day mortality rate was 8.08%, and 90-day mortality rate was 5.05%. In group FFP I, 5 (13.5%) of 37 patients died; in FFP II, 5 (11.9%) of 42 died, and 2 of them were operated on; no patients died in group FFP III, and all of them were operated on; and in group IV, 1 (10%) of 10 died, and this patient was not operated on due to severe comorbidities (Figure 4).

Overall operating rate was 28/99 (28.2%), and only two patients who underwent surgery, qualified due to FFP II, died.

## 4. Discussion

In this study, we analyzed a large cohort of patients suspected of having pelvic fragility fractures who were referred to the aforementioned hospitals between January 2023 and June 2025. Our findings provide valuable insights into the demographic characteristics, types of fractures, and their impact on hemoglobin levels and mortality. Insights were gained from the CT examination. The significance of fragility fractures, particularly in the elderly population, has been well-documented in the previous literature, emphasizing the need for comprehensive management strategies [2,24].

A total of 99 patients were enrolled, among whom 88 (88.9%) were female, and 11 (11.1%) were male participants. The mean age of the patients was 81.12 years (SD = 10.13), indicating that pelvic fractures are prevalent in the elderly population. This aligns with findings from other studies, such as those by Haentjens et al. [25] and Sivapathasuntharam et al. [26], which report similar demographic profiles, emphasizing that women are disproportionately affected by fragility fractures due to factors such as osteoporosis.

The mean duration of hospitalization was 4.86 days (SD = 4.48), which is relatively shorter compared to the findings by Reito et al. [27] and Rommens et al. [20], where the average hospital stay for patients with pelvic fractures was reported to be 11 days. This variability reflects the differences in fracture severity, complications, and chosen method of treatment.

Plain X-rays are usually the primary diagnostic method because they are cost-effective, being both inexpensive and widely accessible. Nevertheless, they pose difficulties in accurately diagnosing fragility fractures of the pelvis (FFPs) due to factors such as reduced bone quality, overlapping visceral shadows, and often minimal initial displacement of the fractures. A study by Mennen al. [18] showed that fractures of the posterior pelvic ring are often missed, with detection rates ranging from 32% to 87%. Kanakaris et al. [28] reported that more than 80% of FFP patients with persistent pelvic pain end up having advanced imaging at some point in their care.

In our study, every patient was examined with CT to assess the posterior pelvic ring, and in 62 (60.6%) cases, the posterior ring was fractured. Putzeys et al. [29] examined 318 fragile patients with CT and posterior ring fractures were observed in 79.4%. Similar to our study are the results presented by Alnaib et al. [30], who detected posterior ring fractures in 61% of patients.

CT imaging seems to be a very valuable and inexpensive tool for detecting posterior ring fractures. Gordon et al. [31] proposed a useful algorithm for detecting posterior ring fractures in elderly patients with persistent posterior pelvic pain. When a fracture is not detected on CT, the authors suggested performing an MRI. Henes et al. [32] compared the diagnostic accuracy of magnetic resonance imaging (MRI) and computed tomography (CT) in the detection of pelvic fractures. MRI proved to be significantly better compared to CT, particularly in the depiction of fractures of the sacrum, reaching a sensitivity of 98.6% compared to 66.1% in CT scans.

According to the aforementioned Fragility Fracture of the Pelvis Classification, in our study, 37 (37.4%) fractures were assessed as type I, 42 (42.4%) as type II, 10 (10.1%) as type III, and 10 (10.1%) as type IV. The fracture type distribution is different from that presented by Banierink et al. [24], where type I was present in 60%, II in 27%, III in 8%, and IV in 5%. In Rommens and Hofmann’s original paper [22], they observed a 51.8% prevalence of type II fractures.

### 4.1. Hemoglobin Levels

The risk of severe bleeding and associated hemodynamic instability, although less frequent in comparison to standard high-energy pelvic injuries (2.4% vs. 40%), should also be considered in patients with FFPs [33,34].

A decrease in hemoglobin levels of more than 2 g% was noted in 20.2% of patients, which may indicate internal bleeding or other serious clinical conditions. The most significant drop in hemoglobin levels was observed in Nakatani II (8.08%) and III (11.1%) groups. There are very few studies concerning hemoglobin levels among fragility fractures of the pelvis, and we agree with the findings presented by Kołodziejczyk et al. [35] that all patients should have a hemoglobin level assessment, which should be repeated after 24 h. According to this study, patients with an initial Hb level of 10 g% should be monitored. In another study, de Herdt et al. [36] revealed that only <1% of patients with FFPs require blood transfusion. Moreover, there is only one case report concerning bleeding in a fragile patient with an isolated pubic ramus fracture in Nakatani II [37].

### 4.2. Mortality

The overall mortality rate in our study was 13.13% (30-day mortality was 8.08% and 90-day mortality was 5.05%). This finding is lower than that presented by Reito et al. [27], who found a 30-day mortality rate of 7.3% and a 90-day mortality rate of 11.4% [27]. One-year mortality rates vary in the literature, from 3.7% reported by Krappinger et al. [38] to 35% reported by Banierink et al. [39].

An interesting finding is that no patients died in group FFP III, and only one did so in group IV, and that patient was not operated on due to severe comorbidities. The other patients who died qualified for group I or II. In group FFP I, 5 (13.5%) of 37 patients died; in FFP II, 5 (11.9%) of 42 died, and 2 of them were operated on; no patients died in group FFP III, and all of them were operated on; and in group IV, 1 (10%) of 10 died. Our findings are similar to those presented in a randomized controlled trial published by Thiesen. In his study, only patients from group FFP II died (39 out of 81) [40].

### 4.3. Limitations

One limitation of our study is the relatively small sample size, particularly in groups III and IV. This may result in selection bias affecting our results. Additionally, the research was conducted across four different hospitals, each of which may employ varying approaches to operative treatment. This variability could introduce potential bias in the observed mortality rates. Another limitation is the lack of functional outcomes, which could be important in future decision making.

Future studies should focus on comparing patients’ functional independence in relation to specific treatment modalities. Such comparisons will be instrumental in guiding decision-making regarding the most appropriate treatment options for particular groups within the Fragility Fracture of the Pelvis (FFP) Classification.

## 5. Conclusions

This study revealed that while plain X-rays could identify only anterior pelvic ring fractures in fragile patients, computed tomography (CT) scans subsequently detected posterior ring fractures in 60.6% of cases. It is worth emphasizing that CT improves detection compared to plain X-rays, not MRI. This underscores the value of CT as a crucial tool for recognizing these life-threatening injuries, specifically fragility fractures of the pelvis.

Patients with Nakatani II and III pelvic ramus fractures exhibited the most significant decreases in hemoglobin levels. Given the potential for substantial drops in hemoglobin, all patients with fragility fractures of the pelvis should be closely monitored for at least one day following admission.

Moreover, the highest mortality rates were observed in the FFP I and II groups, highlighting the increased risk associated with these fracture types.

## Data Availability

Any additional data supporting this study are available from the corresponding author (M.K.) upon reasonable request.
